# Neuromuscular and Kinematic Adaptation in Response to Reactive Balance Training – a Randomized Controlled Study Regarding Fall Prevention

**DOI:** 10.3389/fphys.2018.01075

**Published:** 2018-08-07

**Authors:** Anne Krause, Kathrin Freyler, Albert Gollhofer, Thomas Stocker, Uli Brüderlin, Ralf Colin, Harald Töpfer, Ramona Ritzmann

**Affiliations:** ^1^Department of Sport Science, University of Freiburg, Freiburg, Germany; ^2^Institute of Training and Computer Science in Sport, German Sport University Cologne, Cologne, Germany; ^3^Department of Mechatronics, University of Applied Sciences, Esslingen, Germany

**Keywords:** reflex, electromyography, posture control, balance, conventional balance training, kinematics, lifespan, reaction

## Abstract

Slips and stumbles are main causes of falls and result in serious injuries. Balance training is widely applied for preventing falls across the lifespan. Subdivided into two main intervention types, biomechanical characteristics differ amongst balance interventions tailored to counteract falls: conventional balance training (CBT) referring to a balance task with a static ledger pivoting around the ankle joint versus reactive balance training (RBT) using externally applied perturbations to deteriorate body equilibrium. This study aimed to evaluate the efficacy of reactive, slip-simulating RBT compared to CBT in regard to fall prevention and to detect neuromuscular and kinematic dependencies. In a randomized controlled trial, 38 participants were randomly allocated either to CBT or RBT. To simulate stumbling scenarios, postural responses were assessed to posterior translations in gait and stance perturbation before and after 4 weeks of training. Surface electromyography during short- (SLR), medium- (MLR), and long-latency response of shank and thigh muscles as well as ankle, knee, and hip joint kinematics (amplitudes and velocities) were recorded. Both training modalities revealed reduced angular velocity in the ankle joint (*P* < 0.05) accompanied by increased shank muscle activity in SLR (*P* < 0.05) during marching in place perturbation. During stance perturbation and marching in place perturbation, hip angular velocity was decreased after RBT (*P* from TTEST, *P*_t_ < 0.05) accompanied by enhanced thigh muscle activity (SLR, MLR) after both trainings (*P* < 0.05). Effect sizes were larger for the RBT-group during stance perturbation. Thus, both interventions revealed modified stabilization strategies for reactive balance recovery after surface translations. Characterized by enhanced reflex activity in the leg muscles antagonizing the surface translations, balance training is associated with improved neuromuscular timing and accuracy being relevant for postural control. This may result in more efficient segmental stabilization during fall risk situations, independent of the intervention modality. More pronounced modulations and higher effect sizes after RBT in stance perturbation point toward specificity of training adaptations, with an emphasis on the proximal body segment for RBT. Outcomes underline the benefits of balance training with a clear distinction between RBT and CBT being relevant for training application over the lifespan.

## Introduction

Perturbation-related falls in response to slips or trips are major causes (>60%) of injuries over the lifespan (Gallagher et al., 1984; Winter, 1995; Rubenstein, 2006). As a consequence, affected individuals suffer from physical impairments, reduced autonomy, and a constrained quality of life (Tinetti, 1994; Rubenstein, 2006; Heinrich et al., 2010). Fall scenarios and related injuries among children (Gallagher et al., 1984), adults (Timsina et al., 2017), and seniors (Alexander et al., 1992) constitute a major public health problem and have gained socioeconomic importance due to high clinical and consequential costs (Miller et al., 2000; Stevens et al., 2006). Hence, scientific debates about efficient countermeasures move into focus (Granacher et al., 2011a).

Comparing populations of high-risk fallers to non-fallers, beyond cognitive and strength deficits, factors as the following have been empirically identified as predisposing a person to a greater fall incidence: a decreased ability to stabilize postural equilibrium (Arampatzis et al., 2008), deteriorated balance recovery (van Dieën et al., 2005; Karamanidis and Arampatzis, 2006), undersized timing, and extent of the postural response (Tang and Woollacott, 1998). In particular, distinctly declined neuromuscular activity (Tang and Woollacott, 1998; Granacher and Gollhofer, 2005; Gehring et al., 2014), smaller peak knee displacement after translation (Horak et al., 2005) or rotation of the support surface (Bakker et al., 2006), increased joint torques and angular velocities in gait (Lee and Kerrigan, 1999; Lord et al., 2001; Dobkin and Dobkin, 2003) have been determined in fallers. In other words, not the age itself, but rather the overall level of movement control seems to be the limiting factor to break one’s fall. This can be verified in both children who lack adult-like maturity of their joint control (Ganley and Powers, 2005) and in elderly who lose acquired skills as a result of progressive aging-induced degradation (Sawers et al., 2017). Independent of the age category, fall prevention programs have been established to counteract the falls and diminish consequential costs (Granacher et al., 2011a).

Scientific debates dealing with ***fall prevention*** outlined paradigms, including balance training, to counteract postural instability through more effective compensatory muscle activation in young and old sub-samples (Granacher et al., 2006; Karlsson et al., 2013; Ungar et al., 2013). Recently, a novel type of balance training was introduced: reactive balance training (RBT) is an intervention *simulating* the fall situation itself by the application of unpredictable, random, multi-directional displacements of the support surface (Shumway-Cook et al., 2003; Obuchi et al., 2004; Bieryla and Madigan, 2011; Granacher et al., 2011b, 2012; Mansfield et al., 2015). It was shown that crucial adaptive skills for resisting falls can be acquired rapidly among young and older adults through a single session of RBT with exposure to slips on a movable platform (Bhatt and Pai, 2008; Pai et al., 2010; Bhatt et al., 2011) and transfer effects persist beyond the laboratory for fall situations encountered in daily living (Bhatt et al., 2006). However, evidence for the effectiveness of RBT applied over several weeks is still limited and varies greatly regarding perturbation stimuli in simulated fall risk paradigms (Fitzgerald et al., 2000, 2002; Shumway-Cook et al., 2003; Obuchi et al., 2004; Shimada et al., 2004; Hurd et al., 2006; Mansfield et al., 2010; Bieryla and Madigan, 2011; Bierbaum et al., 2013). Besides its effectiveness, insights into the specific neuromuscular modulations after such forms of balance training are still lacking. Nonetheless, the knowledge about those modulations is the basis to develop further recommendations for RBT as a possible fall avoidance training. Furthermore, fall preventive adaptations are further merely assessed in indirect measures, such as reduced time to stabilize equilibrium (Shumway-Cook et al., 2003; Bieryla and Madigan, 2011) and modified reactions to a stimulus, which are assessed by means of frequency and contact time (Mansfield et al., 2010) concomitant with changed neuromuscular activation for regaining equilibrium (Obuchi et al., 2004). Although conjunctions with fall (Shimada et al., 2004) and injury prevention (Hurd et al., 2006), or even with returning to physical activities within the rehabilitation process are assumed (Fitzgerald et al., 2000, 2002), fundamental evidence about the associated functional benefits and underlying neuromuscular mechanisms for avoidance of falls is missing.

In contrast, conventional balance training (CBT) performed on unstable surfaces and convex devices has been validated to improve postural stability (Granacher et al., 2006; Gruber et al., 2007a,b; Taube et al., 2008; Freyler et al., 2014) and to elicit functional and neuromuscular adaptations beneficial for fall avoidance, such as augmented muscle strength (Bruhn et al., 2006). This includes explosive force and rate of force development (Gruber and Gollhofer, 2004; Taube et al., 2007) as well as modified muscle activity, such as increased activation or reduced co-contraction by means of improved muscle coordination, induced by neural adaptations within the central nervous system (Granacher et al., 2009; Nagai et al., 2012; Oliveira et al., 2013; Behrens et al., 2015). These neuronal adaptations were specified by higher amplitudes and shorter latencies in the early reflex responses in the shank muscles, resulting in augmented ankle joint stiffness (Granacher et al., 2006) and reduced fall frequency (Madureira et al., 2007) in response to postural perturbations.

**FIGURE 1:**
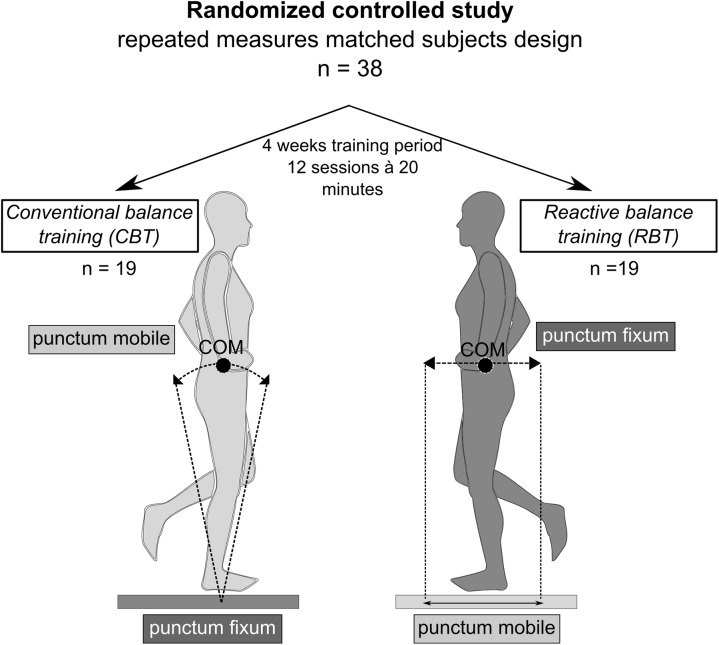
Study overview and models comparing conventional versus reactive balance training. Comparison of the two training paradigms on the basis of pendulum models (Winter, 1995; Schmitt, 2003) with the conventional training (CBT) describing a single inverted pendulum, while participants in the reactive balance training (RBT) group oscillate around the fixation point located within the center of gravity. In this randomized controlled study, baseline data collection was followed by a random “matched-pair” assignment of 39 volunteers to either the CBT-group or RBT-group. Before and after 4 weeks of training, measurements were conducted to assess changes in fall-related risk factors.

Comparing RBT to CBT from a biomechanical point of view, stabilizing torques are shifted from distal to proximal. According to the pendulum model (Winter, 1995; Schmitt, 2003), “punctum fixum” and “punctum mobile” are exchanged from the support surface (CBT) to the center of mass itself (RBT), challenging the subject to maintain equilibrium above an unstable moving support surface (RBT) instead of oscillating around a fixed ledger (CBT) (Maki and McIlroy, 2006) (**Figure 1**). As a consequence, RBT requires an accurate repositioning of the center of mass (COM) utilizing rapid and appropriate neuromuscular responses to regain a stable body position after surface translation. Thus, RBT may challenge reactive postural stability more than CBT (Horstmann and Dietz, 1990; Yim-Chiplis and Talbot, 2000) and may be more effective as an intervention to counteract falls (Freyler et al., 2016).

Based on the aspects mentioned above, the rationale of the study was to compare neuromuscular and kinematic adaptations of RBT and CBT in terms of the relevant fall risk factors. Adaptations were measured during functional tasks of stance and marching in place perturbation before and after the training intervention. Focus was set on muscular activation patterns characterized by phase-specific reflex parameters indicated as short- (SLR), medium- (MLR), and long- (LLR) latency responses following the onset of perturbation (Horak and Nashner, 1986; Diener et al., 1988; Gollhofer and Rapp, 1993). While SLR mainly comprises Ia afferent reflex responses, information during MLR is also transmitted via the midbrain and brainstem. Latest responses (LLR) encompass transcortical pathways through the cerebral cortex (Jacobs and Horak, 2007). RBT might simulate a fall risk situation, which is why differentiated neuromuscular and kinematic effects were expected to be more pronounced in RBT than CBT. It was hypothesized that improvements induced by RBT in dynamic stabilization after perturbation would ameliorate neuromuscular control for slip recovery characteristics, comprising (a) an elevated reflex activity in the shank and thigh muscles and (b) enhanced kinematic segmental stabilization to compensate for the disturbing stimulus during stance and marching in place perturbation.

## Materials and Methods

### Participants

Thirty-nine healthy participants of sport students [24 females (*f*), 15 males (*m*), age 24 ± 3 years] participated in the study. The sample size was estimated by means of a power analysis (test attributes: *F*-test, repeated-measures analysis of variance, within-between factors, *f* = 0.25, medium effect; alpha = 0.05; Power = 0.75) (Faul et al., 2007). Volunteers who performed any other kind of balance training or suffering from acute injuries or neurological irregularities were excluded. We requested a written document from all subjects confirming the absence from any kind of additional balance training apart from this study. All participants, furthermore, gave written informed consent to the experimental procedure, which is approved by the ethics committee of the University of Freiburg (EK Freiburg 16/13) in accordance with the latest revision of the Declaration of Helsinki. Using the concealed allocation procedure, participants were randomly divided up by “matched-pairs” either into a RBT group (RBT-group, 11f/8m, age 24 ± 3 years, height 173 ± 7 cm, weight 67 ± 12 kg) or a control group that performed conventional balance training (CBT-group, 13f/7m, age 24 ± 3 years, height 172 ± 9 cm, weight 67 ± 10 kg, one male drop-out, rate 2.6%). Matched-pairs were determined prior to the interventions based on the postural sway measures (described in detail in the outcome measures). Therefore, subjects who demonstrated almost equivalent performance levels prior to training were randomly allocated either to the CBT-group or to the RBT-group by drawing lots.

### Experimental Design

**FIGURE 2:**
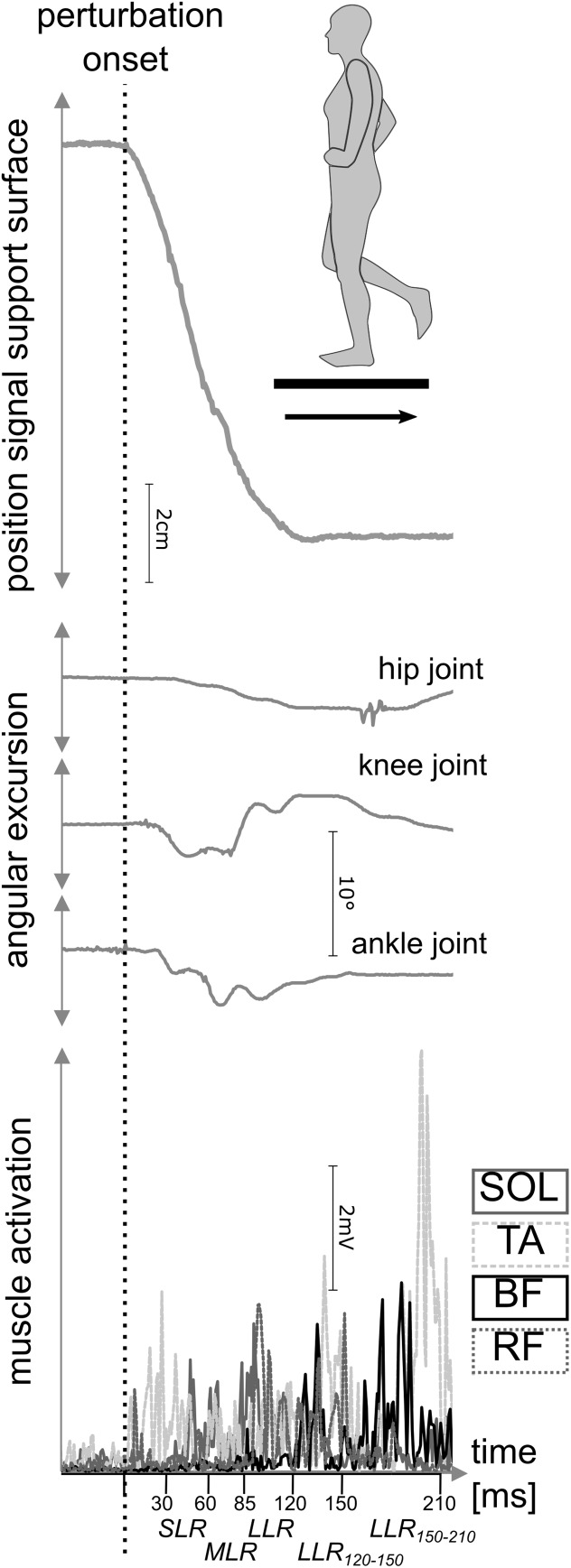
Outcome measures during posterior perturbation of a representative participant. Recording of neuromuscular data (bottom) and kinematic data (middle) during posterior translation of the support surface (top) were carried out. For muscular activation, the soleus (SOL), tibialis anterior (TA), biceps femoris (BF), and rectus femoris (RF) muscles were measured during short- (SLR), medium- (MLR), and long-latency responses (LLR) after perturbation onset. Simultaneously, joint excursions of the ankle, knee, and hip joint were assessed.

In a randomized control trial, a repeated-measures matched-subject design (subjects and therapists were not blinded; assessors were blinded) was used to ascertain the effect of a 4-week trial of RBT versus CBT on neuromuscular and biomechanical aspects of fall avoidance characteristics, postural reflexes and kinematics in response to perturbation (**Figure 2**). Two protocols were used in a randomized order to assess the ability to compensate for stance and gait disturbances in stumbling situations after the interventions. However, for pre to post measurements, we conducted the same order in each individual to exclude any effects due to preceding tasks. In *protocol 1*, training-induced effects on postural reactions in a static balance setting was investigated, while, in *protocol 2*, a dynamic test setting during locomotion was investigated. Electromyograms (EMGs) of four leg muscles as well as ankle, knee, and hip joint kinematics were recorded during both settings before and after the interventions (**Figure 2**). Prior to each measurement, isometric maximal voluntary contractions were performed for all recorded muscles according to Freyler et al. (2016) for EMG normalization. Training and testing sessions were surveyed, supervised, and documented by the authors.

### Training Intervention

For both groups the training interventions lasted 4 weeks and comprised three sessions per week lasting 20 min each. Groups trained in parallel. One session consisted of four parts separated by 1-min breaks, each part comprised four repetitions, respectively. Each repetition lasted 1 min and was divided into 40 s training with a 20 s intermittent break (Taube et al., 2008; Lesinski et al., 2015). Training settings were matched regarding training frequency, number and duration of sets and pauses. RBT was performed on an electromagnetically driven swinging platform (Perturmed, Brüderlin, Germany) generating surface translations in the horizontal plane (eight directions: medio-lateral (ml), antero-posterior (ap), and the diagonals (for a detailed description of the device see Freyler et al., 2015). Meanwhile, the CBT-group trained with conventional balance devices, including unstable surfaces ranging from easy (Airex^®^ Balance-Pad) to more challenging postural demands (Togu^®^ Dynair air cushions ø 33 cm, Aero-Step cushions 51 cm × 37 cm × 8 cm/46 cm × 32 cm × 8 cm, Jumper^®^ 52 cm × 24 cm) of different balance performance complexities (Gruber et al., 2007a; Taube et al., 2008; Lesinski et al., 2015). For both groups, participants were instructed to maintain or to recover stability with their head forward-facing, arms akimbo and the non-standing leg being flexed. If this task was accomplished easily, the level of difficulty was raised successively and individually within the training period. For RBT, the perturbation program (embracing 16 levels in total) was increased in difficulty in the following order: increase of translation displacement (2–4–6 cm), increase of additional directions (ap–ml– diagonals), and reduction of durations between perturbations (4–2–1 s break). For CBT, support surfaces were varied for an individual increase of difficulty. Subsequently, eyes were closed during training to exclude visual cues for RBT and CBT.

### Protocols

To exclude habituation effects prior to measurements, subjects practiced for a period of 10 min in the test conditions. The order of balance protocols and tasks was randomized (by drawing lots) to exclude confounding effects but was controlled in post-assessments referring to pre-measurements.

#### Protocol 1 – Stance Perturbation

To determine compensatory reactive responses to sudden perturbations, as they occur during stumbling, posterior translations of the support surface were induced randomly to the left leg during static monopedal stance (**Figure 2**). Translations were conducted on a customized platform [Department of Sport Science, University of Freiburg, cf. (Mornieux et al., 2014)] moving horizontally backward with an amplitude of 16.21 ± 0.04 cm and impulse duration of 140 ± 3 ms resulting in a mean velocity of 1.22 ± 0.02 m ⋅ s^-1^ and a maximal acceleration of 11.2 ± 0.5 m ⋅ s^-2^. Fifteen perturbations were induced randomly within a range of 2–4 s (Hall and Jensen, 2002). Participants were asked to sustain balance on one leg (right) and to stabilize equilibrium as quickly as possible. In case of balance loss or using their other leg, trials were repeated.

#### Protocol 2 – Marching in Place Perturbation

For functional testing, participants marched in place with the left foot stepping on the movable platform. Fifteen displacements were induced randomly after trespassing a light barrier so that the support surface was translated during full weighted foot-contact unexpected in time (Mornieux et al., 2014). Gait pace was controlled and standardized by a metronome (112 beats ⋅ min^-1^). Prior to recording, all participants practiced the task for 10 min so that participants were habituated to the task and marching steps were reliable. Arm were moved in a crisscrossing pattern, and vision was forward-facing. Translations were induced by the same customized platform as described in *protocol 1* with an amplitude of 20.05 ± 0.05 cm and impulse duration of 150 ± 2 ms resulting in a mean velocity of 1.34 ± 0.02 m ⋅ s^-1^ and a maximal acceleration of 14.2 ± 0.8 m ⋅ s^-2^ (Granacher et al., 2006; Mornieux et al., 2014). Participants were asked to sustain balance after perturbation and to stabilize equilibrium as quickly as possible. In case of balance loss, defined as bracing oneself against the wall next to the customized platform, trials were repeated.

### Outcome Measures

#### Neuromuscular Data

According to SENIAM (Hermens et al., 2000), surface EMGs of selected muscles of the shank and the thigh of the left leg [soleus (SOL), tibialis anterior (TA), biceps femoris (BF), and rectus femoris (RF)] were recorded. Bipolar Ag/AgCl surface electrodes (Ambu Blue Sensor P, Ballerup, Denmark; diameter 9 mm, center-to-center distance 25 mm) were placed onto the muscle belly in line with the underlying muscle fibers. A reference electrode was fixed onto the patella. Interelectrode resistance was kept below 5 kΩ by means of shaving, light abrasion, and degreasing of the skin with a disinfectant. EMG signals were transferred via shielded cables to an amplifier (band-pass filter 10 Hz–1 kHz, 1000× amplified) and sampled with 1000 Hz.

#### Kinematics

Ankle, knee, and hip joint angles were recorded with monoaxial electrogoniometers (Biometrics^®^, Gwent, United Kingdom). For that purpose, the center of rotation of the goniometer was placed over the rotational axis of the respective joint (ankle: malleolus lateralis, knee: knee joint cavity, and hip: trochanter major) and the two arms (proximal and distal) were aligned in extension of the joint axes (ankle: pointing toward fifth metatarsal and longitudinal axis of the shank, knee: pointing toward malleolus lateralis and trochanter major, and hip: longitudinal axis of the femur and trunk). For details, see Freyler et al. (2016).

### Data Processing

For the analysis of neuromuscular data, the EMG of each muscle was rectified, averaged, and integrated [iEMG (mVs)]. iEMG was analyzed regarding the reflex phases after perturbation: SLR (30–60 ms), MLR (60–85 ms), and LLR (85–120 ms) (Grey et al., 2001; Taube, 2006). In addition, the iEMG was calculated for the interval 120 ms until the end of the perturbation (150 ms) and according to Dietz et al. (1989) up to 210 ms after the perturbation onset. Subsequently, all iEMG data were individually normalized to those recorded during maximal voluntary contraction (Halaki and Gi, 2012). An onset latency of each muscle was identified as the first burst >2 standard deviations above the baseline iEMG (Henry et al., 1998). Percentage differences (pre/post) were calculated from normalized values corresponding to baseline data for each participant and subsequently averaged.

Ankle, knee, and hip joint kinematics were expressed as mean joint amplitudes in the time interval during posterior translation [°]. Angular excursion was averaged for each participant and normalized to the neutral position defined at 90° in the ankle joint (longitudinal axis foot/fibula) and 180° in the knee (longitudinal axis fibula/femur) and hip joint (longitudinal axis femur/trunk). The angular velocity of joint excursions (Ω) was assessed as follows: Ω = *x* ⋅*t*^-1^ with *x* describing the displacement [°] and *t* the time to maximal excursion [s] in a timeframe of 0–200 ms.

### Statistics

To determine statistical differences within the independent variable groups (2, RBT-group versus CBT-group) and time (2, pre versus post) a repeated-measures analysis of variance (rmANOVA) was conducted. Dependent variables were iEMG data (SOL, TA, BF, RF), latencies (SOL, BF, RF) and angular excursion and velocity (ankle, knee, and hip joint). The normality of the data was evaluated with the Kolmogorov–Smirnov test; data followed a normal distribution. If the assumption of sphericity measured by Mauchly’s sphericity test was violated, Greenhouse-Geisser correction was used. To detect one-sided effects, additional one-tailed paired student’s *t*-tests (TTESTs) were calculated. The level of significance was defined at *P* < 0.05. “*P*” indicates the level of significance for rmANOVA, “*P*_t_” describes results of TTESTs. To control for changes in onset latency within the different muscles, a univariate ANOVA was conducted including *post hoc* tests.

Additionally, effect sizes were calculated according to Cohen (*d*) and by means of Partial Eta squared ($\eta_{p}^{2}$, see **Tables 1**–**5**). Reference values are defined as trivial with *d* < 0.2 ($\eta_{p}^{2}$ < 0.01), as small with 0.2 < *d* > 0.5 (0.01 < $\eta_{p}^{2}$ > 0.06), as medium with 0.5 < *d* > 0.8 (0.06 < $\eta_{p}^{2}$ > 0.14) and as large effects with *d* > 0.8 ($\eta_{p}^{2}$ > 0.14) (Cohen, 1988, 1992; Thalheimer and Cook, 2002).

**Table 1:** Neuromuscular data during stance perturbation.

	Protocol 1: Stance perturbation					
	
	Group	Δ pre/post	*P*_t_	*d*	*P*	$\eta_{p}^{2}$
SLR iEMG [%]					
RF	RBT	**+60 ± 93**	**0.01**	0.95	*F*(1,16) = 5.390, *p* = **0.034**	0.252
	CBT	+46 ± 120	0.06	0.55		
BF	RBT	+88 ± 209	0.06	0.62	*F*(1,14) = 3.846, *p* = 0.070	0.216
	CBT	+45 ± 198	0.19	0.33		
TA	RBT	+1 ± 60	0.48	0.02	*F*(1,13) = 0.057, *p* = 0.815	0.004
	CBT	-5 ± 31	0.25	0.23		
SOL	RBT	-11 ± 39	0.14	0.40	*F*(1,16) = 0.038, *p* = 0.847	0.002
	CBT	+11 ± 57	0.20	0.29		
MLR iEMG [%]					
RF	RBT	+29 ± 70	0.05	0.60	*F*(1,16) = 5.892, *p* = **0.027**	0.269
	CBT	+22 ± 67	0.09	0.47		
BF	RBT	+62 ± 182	0.11	0.50	*F*(1,13) = 1.299, *p* = 0.275	0.091
	CBT	-6 ± 35	0.25	0.26		
TA	RBT	+12 ± 61	0.23	0.30	*F*(1,13) = 0.614, *p* = 0.447	0.045
	CBT	-3 ± 36	0.35	0.13		
SOL	RBT	-9 ± 45	0.21	0.29	*F*(1,16) = 0.006, *p* = 0.939	<0.001
	CBT	+5 ± 48	0.34	0.15		
LLR iEMG [%]					
RF	RBT	+1 ± 55	0.48	0.02	*F*(1,16) = 2.194, *p* = 0.158	0.121
	CBT	+32 ± 82	0.06	0.56		
BF	RBT	+42 ± 97	0.06	0.64	*F*(1,13) = 1.646, *p* = 0.222	0.112
	CBT	+39 ± 221	0.25	0.25		
TA	RBT	+26 ± 159	0.27	0.24	*F*(1,14) = 0.361, *p* = 0.558	0.025
	CBT	0 ± 43	0.49	0.01		
SOL	RBT	-10 ± 46	0.20	0.30	*F*(1,16) = 0.544, *p* = 0.471	0.033
	CBT	+26 ± 113	0.16	0.34		
LLR_120-150_ iEMG [%]					
RF	RBT	**-17 ± 39**	**0.03**	0.65	*F*(1,15) = 4.607, ***p* = 0.049**	0.235
	CBT	**+134 ± 218**	**0.01**	0.90		
BF	RBT	**+123 ± 157**	**<0.01**	1.14	*F*(1,16) = 10.713, ***p* = 0.005**	0.401
	CBT	**+39 ± 72**	**0.02**	0.79		
TA	RBT	**+94 ± 129**	**<0.01**	1.06	*F*(1,17) = 8.572, *p* = 0.009	0.335
	CBT	+6 ± 114	0.41	0.08		
SOL	RBT	**+97 ± 105**	**<0.01**	1.35	*F*(1,17) = 21.366, ***p* < 0.001**	0.557
	CBT	**+52 ± 80**	**<0.01**	0.95		
LLR_150-210_ iEMG [%]					
RF	RBT	+27 ± 101	0.13	0.39	*F*(1,17) = 8.464, ***p* = 0.010**	0.332
	CBT	**+96 ± 140**	**<0.01**	1.00		
BF	RBT	+50 ± 159	0.09	0.46	*F*(1,16) = 2.704, *p* = 0.120	0.145
	CBT	**+31 ± 58**	**0.02**	0.77		
TA	RBT	+14 ± 187	0.38	0.11	*F*(1,17) = 2.502, *p* = 0.132	0.128
	CBT	**+62 ± 81**	**<0.01**	1.11		
SOL	RBT	**+91 ± 93**	**<0.01**	1.42	*F*(1,17) = 19.998, *p* < **0.001**	0.541
	CBT	**+125 ± 196**	**<0.01**	0.93		


*Mean changes in iEMG (%) in the short- (SLR), medium- (MLR), and long-latency responses (LLR) after perturbations during quiet monopedal stance (protocol 1). iEMG data are normalized to baseline values. Significant changes in response to the training intervention (TTEST, *P_*t*_*) with effect sizes (Cohen’s d, *d*) as well as time interactions (rmANOVA, *P*) with effect sizes (Partial Eta Squared, $\eta_{p}^{2}$ ) are illustrated in bold in the right columns with *P_*t*_* and *P* < 0.05.*

**Table 2:** Onset latency of the electromyograms during stance and marching in place perturbation.

	Group	Δ pre/post [%]	*P*_t_	*d*	*P*	$\eta_{p}^{2}$
Protocol 1: Stance perturbation					
RF	RBT	-**16 ± 13**	**<0.01**	1.70	*F*(1,17) = 7.852, *p* = 0**.012**	0.316
	CBT	+1 ± 23	0.44	0.05		
BF	RBT	-**11 ± 10**	**<0.01**	1.60	*F*(1,17) = 0.151, *p* = 0.703	0.009
	CBT	+8 ± 31	0.15	0.36		
SOL	RBT	-**5 ± 9**	**0.02**	0.75	*F*(1,17) = 18.478, *p* **< 0.001**	0.521
	CBT	-**12 ± 12**	**<0.01**	1.44		
Protocol 2: Marching in place perturbation					
RF	RBT	-**14 ± 9**	**<0.01**	2.22	*F*(1,17) = 4.621, ***p* = 0.046**	0.214
	CBT	+1 ± 25	0.45	0.04		
BF	RBT	-**12 ± 14**	**<0.01**	1.25	*F*(1,16) = 2.436, *p* = 0.138	0.132
	CBT	+8 ± 22	0.06	0.55		
SOL	RBT	-2 ± 8	0.17	0.33	*F*(1,17) = 0.004, *p* = 0.947	<0.001
	CBT	+2 ± 18	0.31	0.17		


*Mean changes from pre to post in EMG onset latency (%) for the protocols 1 and 2. iEMG data are normalized to baseline values. Significant changes in response to the training intervention (TTEST, *P_*t*_*) with effect sizes (Cohen’s d, *d*) as well as time interactions (rmANOVA, *P*) with effect sizes (Partial Eta Squared, $\eta_{p}^{2}$ ) are illustrated in *bold* in the right columns with P_*t*_ and *P* < 0.05.*

**Table 3:** Kinematic data during stance perturbation.

	Protocol 1: Stance perturbation					
	
	Group	Δ pre/post	*P*_t_	*d*	*P*	$\eta_{p}^{2}$
Amplitude [°]					
hip	RBT	-0.45 ± 1.39	0.14	0.42	*F*(1,11) = 0.066, *p* = 0.802	0.006
	CBT	+0.18 ± 1.25	0.28	0.18		
knee	RBT	+0.63 ± 1.49	0.05	0.51	*F*(1,16) = 6.368, *p* = **0.023**	0.285
	CBT	**+0.46 ± 1.11**	**0.04**	0.47		
ankle	RBT	+0.91 ± 4.26	0.20	0.25	*F*(1,16) = 1.493, *p* = 0.239	0.085
	CBT	+0.82 ± 4.06	0.20	0.27		
Velocity [degrees ⋅ s^-1^]					
hip	RBT	-**6.66 ± 11.34**	**0.04**	0.65	*F*(1,10) = 0.918, *p* = 0.360	0.084
	CBT	+1.87 ± 13.77	0.29	0.18		
knee	RBT	-2.19 ± 23.29	0.35	0.16	*F*(1,16) = 0.180, *p* = 0.677	0.011
	CBT	+0.77 ± 22.22	0.44	0.05		
ankle	RBT	-10.26 ± 23.71	0.06	0.52	*F*(1,14) = 4.627, *p* = **0.049**	0.248
	CBT	-3.88 ± 15.72	0.17	0.25		


*Depicted are averaged changes of amplitude (°) and velocity (degrees ⋅ s^-*1*^) of the ankle, knee, and hip joint excursions after perturbations during quiet monopedal stance (protocol 1). Significant changes in response to the training intervention (TTEST, *P_*t*_*) with effect sizes (Cohen’s d, *d*) as well as time interactions (rmANOVA, *P*) with effect sizes (Partial Eta Squared, **$\eta_{p}^{2}$** ) are illustrated in bold in the right columns with *P_*t*_* and *P* < 0.05.*

**Table 4:** Neuromuscular data during marching in place perturbation.

	Protocol 2: Marching in place perturbation					
	
	Group	Δ pre/post	*P*_t_	*d*	*P*	$\eta_{p}^{2}$
SLR iEMG [%]					
RF	RBT	**+43 ± 84**	**0.02**	0.75	*F*(1,17) = 7.853, *p* = **0.012**	0.316
	CBT	**+80 ± 141**	**0.01**	0.83		
BF	RBT	+59 ± 200	0.13	0.43	*F*(1,14) = 2.624, *p* = 0.128	0.158
	CBT	+53 ± 220	0.18	0.35		
TA	RBT	-8 ± 37	0.21	0.30	*F*(1,15) = 0.438, *p* = 0.518	0.028
	CBT	+14 ± 54	0.14	0.37		
SOL	RBT	+46 ± 119	0.06	0.56	*F*(1,17) = 9.339, *p* = **0.007**	0.355
	CBT	**+52 ± 83**	**0.01**	0.90		
MLR iEMG [%]					
RF	RBT	**+26 ± 45**	**0.02**	0.82	*F*(1,16) = 6.968, *p* = **0.018**	0.303
	CBT	**+42 ± 95**	**0.04**	0.64		
BF	RBT	+38 ± 219	0.26	0.25	*F*(1,13) = 0.441, *p* = 0.518	0.033
	CBT	-1 ± 32	0.47	0.03		
TA	RBT	+9 ± 56	0.27	0.23	*F*(1,15) = 2.024, *p* = 0.175	0.119
	CBT	+10 ± 47	0.19	0.31		
SOL	RBT	+38 ± 113	0.09	0.49	*F*(1,17) = 2.938, *p* = 0.105	0.147
	CBT	+36 ± 108	0.09	0.49		
LLR iEMG [%]					
RF	RBT	-12 ± 39	0.12	0.44	*F*(1,14) = 0.086, *p* = 0.773	0.006
	CBT	+15 ± 78	0.24	0.28		
BF	RBT	+66 ± 212	0.11	0.46	*F*(1,14) = 1.941, *p* = 0.185	0.122
	CBT	+14 ± 124	0.34	0.16		
TA	RBT	+27 ± 98	0.14	0.40	*F*(1,16) = 0.828, *p* = 0.376	0.049
	CBT	-5 ± 32	0.26	0.22		
SOL	RBT	+5 ± 76	0.40	0.09	*F*(1,16) = 2.537, *p* = 0.131	0.137
	CBT	+59 ± 145	0.05	0.59		
LLR_120-150_ iEMG [%]					
RF	RBT	**+85 ± 131**	**<0.01**	0.94	*F*(1,17) = 7.146, *p* = **0.016**	0.296
	CBT	+48 ± 189	0.15	0.37		
BF	RBT	**+185 ± 215**	**<0.01**	1.26	*F*(1,16) = 10.519, *p* = **0.005**	0.397
	CBT	-1 ± 158	0.49	0.01		
TA	RBT	+41 ± 256	0.24	0.24	*F*(1,17) = 5.392, *p* = 0.033	0.241
	CBT	**+86 ± 125**	**<0.01**	1.00		
SOL	RBT	**+96 ± 108**	**<0.01**	1.28	*F*(1,17) = 23.150, *p* < **0.001**	0.577
	CBT	**+110 ± 166**	**<0.01**	0.96		
LLR_150-210_ iEMG [%]					
RF	RBT	**+50 ± 104**	**0.03**	0.69	*F*(1,17) = 5.410, *p* = **0.033**	0.241
	CBT	**+122 ± 261**	**0.03**	0.68		
BF	RBT	**+123 ± 214**	**0.01**	0.84	*F*(1,15) = 5.861, *p* = **0.029**	0.281
	CBT	+53 ± 139	0.08	0.55		
TA	RBT	+86 ± 258	0.09	0.48	*F*(1,17) = 4.879, *p* = **0.041**	0.223
	CBT	+46 ± 125	0.07	0.54		
SOL	RBT	**+89 ± 94**	**<0.01**	1.37	*F*(1,17) = 9.773, *p* = **0.006**	0.365
	CBT	+64 ± 190	0.08	0.49		


*Mean changes in iEMG (%) in the short- (SLR), medium- (MLR), and long-latency responses (LLR) after perturbations during marching in place (protocol 1). iEMG data are normalized to baseline values. Significant changes in response to the training intervention (TTEST, *P_*t*_*) with effect sizes (Cohen’s d, *d*) as well as time interactions (rmANOVA, *P*) with effect sizes (Partial Eta Squared, **$\eta_{p}^{2}$** ) are illustrated in bold in the right columns with *P_*t*_* and *P* < 0.05.*

**Table 5:** Kinematic data during marching in place perturbation.

	Protocol 2: Marching in place perturbation					
	**Group**	**Δ pre/post**	***P*_t_**	***d***	***P***	**$\eta_{p}^{2}$**
	
Amplitude [°]					

Hip	RBT	+0.02 ± 1.74	0.48	0.02	*F*(1,13) = 0.023, *p* = 0.881	0.002
	CBT	+0.03 ± 1.93	0.48	0.02		
knee	RBT	+0.46 ± 1.39	0.09	0.34	*F*(1,13) = 0.087, *p* = 0.773	0.007
	CBT	-0.11 ± 1.17	0.36	0.09		
ankle	RBT	-0.55 ± 1.35	0.06	0.43	*F*(1,15) = 1.729, *p* = 0.208	0.103
	CBT	-0.07 ± 0.94	0.39	0.05		
Velocity [degrees ⋅ s^-1^]					
Hip	RBT	-**5.98 ± 12.44**	**0.05**	0.50	*F*(1,13) = 1.026, *p* = 0.330	0.073
	CBT	-0.33 ± 15.13	0.47	0.03		
knee	RBT	-5.32 ± 23.38	0.18	0.27	*F*(1,10) = 1.179, *p* = 0.303	0.105
	CBT	+2.25 ± 20.31	0.36	0.11		
ankle	RBT	-**12.13 ± 16.00**	**<0.01**	0.99	*F*(1,14) = 6.074, *p* = **0.027**	0.303
	CBT	-4.16 ± 13.85	0.13	0.26		


*Depicted are averaged changes of amplitude (°) and velocity (degrees ⋅ s^-*1*^) of the ankle, knee, and hip joint excursions after perturbations during marching in place perturbation (protocol 2). Significant changes in response to the training intervention (TTEST, *P_*t*_*) with effect sizes (Cohen’s d, *d*) as well as time interactions (rmANOVA, *P*) with effect sizes (Partial Eta Squared, $\eta_{p}^{2}$) are illustrated in bold in the right columns with *P_*t*_* and *P* < 0.05.*

Statistical methods were conducted with the statistics software SPSS 20.0 (SPSS, Inc., Chicago, IL, United States). Group data are presented as mean value ± standard deviation.

## Results

### Protocol 1 – Stance Perturbation

#### Neuromuscular Activity

**FIGURE 3:**
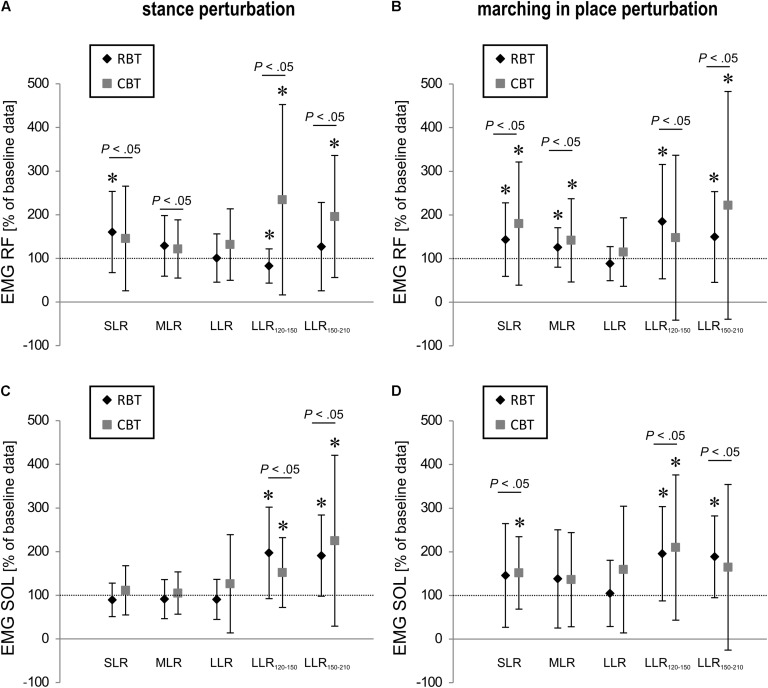
Neuromuscular data of leg muscle activity. Changes of iEMG (in %) of the RF and soleus (SOL) muscles in the short- (SLR), medium- (MLR), LLR as well as 120–150 ms (LLR_120-150_) and 150–210 ms (LLR_150-210_) after stance perturbation **(A,C)** and after marching in place perturbation **(B,D)** are shown. Data are normalized to baseline values (the horizontal dashed line marks baseline values obtained before the interventions). Mean values are illustrated for both training groups – the RBT group and the CBT group. Significant pre/post differences are marked with ^∗^ (*P*_t_ < 0.05), while significant time interactions are illustrated with bars (*P* < 0.05).

Grand means of the iEMG activity are listed in **Table 1** and illustrated in **Figure 3** (for coefficient of variation cf. **Supplementary Table S1**). Significant time effects for both groups (*P* < 0.05) could be shown for RF in SLR and MLR, indicating higher activation amplitude after the training intervention. For the RBT-group, reflexive BF muscle activity showed a tendency toward augmentation in SLR and LLR (*P*_t_ = 0.06). In later time frames of the neuromuscular response, iEMG was enhanced for BF (LLR_120-150_) and for SOL for both groups, RBT and CBT (LLR_120-150_, LLR_150-210_, *P* < 0.05). TA was only increased in LLR_120-150_ (*P*_t_ < 0.05) after RBT and in LLR_150-210_ after CBT (*P*_t_ < 0.05, **Table 1**). Interaction effects (time × group) were observed for SOL, TA, and RF in LLR_120-150_ [SOL *F*(1,17) = 4.85, *P* < 0.05, TA *F*(1,17) = 5.48, *P* < 0.05, RF *F*(1,16) = 10.71, *P* < 0.05]. Effect sizes varied between trivial up to large effect sizes (**Table 1**).

#### Muscle Onset Latencies

Onset latencies diminished significantly for the stance perturbations in RF, BF, and SOL in the RBT-group and in SOL for the CBT-group (**Table 2**, for absolute values cf. **Supplementary Table S2**).

#### Kinematics

**FIGURE 4:**
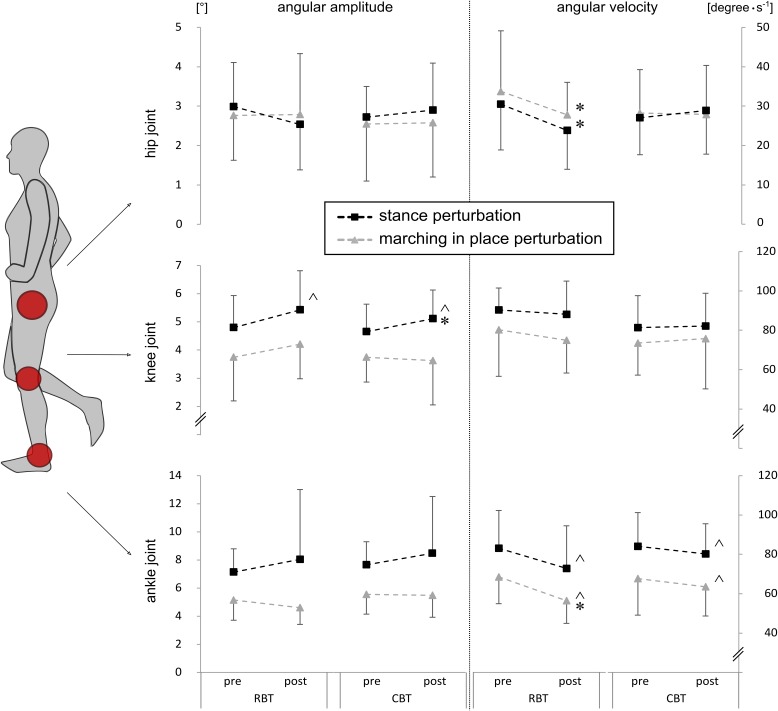
Kinematic data of lower limb joints. Joint kinematics, such as amplitude [°] (left column) and velocity (degrees ⋅ s**^-^**^1^) (right column), for the ankle, knee, and hip joint during stance perturbation (black rectangles, *protocol 1*) and marching in place perturbation (gray triangles, *protocol 2*) are shown. Mean values are illustrated for both training groups – the RBT group and the CBT group. Significant time effects are marked with ˆ (*P* < 0.05) and significant group comparisons (TTESTs) with ^∗^ (*P*_t_ < 0.05).

Grand means of the ankle, knee, and hip joint kinematics are displayed in **Table 3** and illustrated in **Figure 4**. The rmANOVA revealed significant time effects: ankle angular velocity decreased (*P* < 0.05), whereas knee joint amplitude increased for both groups in response to training (*P* < 0.05). Additionally, hip angular velocity was reduced after RBT only (*P*_t_ < 0.05).

Significant percentage changes in neuromuscular and kinematic data were more pronounced after RBT compared to CBT, which is illustrated in larger effect sizes ranging between medium to large sizes for RBT and small to medium sizes for CBT (Cohen’s *d*, cf. **Table 3**).

### Protocol 2 – Marching in Place Perturbation

#### Neuromuscular Activity

Changes in iEMG activity in response to training are displayed in **Table 4** and illustrated in **Figure 3** (for coefficient of variation cf. **Supplementary Table S1**). The rmANOVA revealed significant time effects for both groups: SOL iEMG activity increased in SLR only, while RF iEMG activity increased in SLR and MLR (*P* < 0.05). For RBT, muscle activity of RF, BF, and SOL was enhanced in LLR_120-150_ and LLR_150-210_ significantly (*P* < 0.05). For CBT, shank muscle activity increased significantly in LLR_120-150_ and showed a tendency in LLR_150-210_, in the thigh, muscle responses increased in RF, only (*P*_t_ < 0.05, **Table 4**). Interaction effects (time × group) were observed for BF in LLR_120-150_ [BF *F*(1,16) = 6.77, *P* < 0.05].

Larger effect sizes were reached for CBT versus RBT in the SLR (large versus medium effect sizes). In the MLR, greater effect sizes were evident for RBT (medium to large effect sizes) (**Table 4**). In LLR_120-150_, in almost all muscles, effect sizes reached large effects; in LLR_150-210_ this is still true for SOL for RBT (**Table 4**).

#### Muscle Onset Latencies

Onset latencies diminished significantly in RF and BF in the RBT-group (**Table 2**). No significant changes were manifested for the CBT group.

#### Kinematics

**Table 5** and **Figure 4** display grand means of the ankle, knee, and hip joint kinematics. The rmANOVA revealed a significant time effect illustrated in reduced ankle angular velocity for both groups (*P* < 0.05). For the RBT-group, hip angular velocity also decreased (*P*_t_ < 0.05).

Effect sizes of significant percentage changes ranged from medium to large for RBT and from small to large for CBT.

## Discussion

The purpose of the study was to identify differences between RBT and CBT and to clarify whether RBT improves kinematic and neuromuscular responses associated with the balance recovery after slips. The outcomes of this study outline ***reflex phase- and segment-specific adaptations*** dependent on the context of the movement task: (a) a facilitation of neuromuscular activation in shank and thigh muscles was accompanied by (b) a reduced muscle onset latency and (c) a decline in angular velocity for the hip and ankle joint. Even though no interaction effects could be observed comparing RBT to CBT for the early reflex phases, effect sizes of RBT were more pronounced for recovery response adaptations with a greater emphasis on the proximal body segment. Thus, our hypotheses are verified with the constraint that modified adaptations occur after both trainings.

Two aspects may be of considerable importance for the interpretation of these findings (Gallagher et al., 1984; Alexander et al., 1992; Timsina et al., 2017): the first one deals with training-induced *neuromuscular enhancement* (Hatzitaki et al., 2005; Granacher et al., 2006) and the second with *multi-segmental joint kinematic* associated with the recovery response for fall avoidance (Cham and Redfern, 2001; Bhatt et al., 2006).

### Neuromuscular Enhancement

Both training modalities achieved an enhancement in iEMG activity concomitant with reduced onset latencies in relevant sets of muscles that antagonize the perturbation stimulus. Thereby, marching in place perturbation were compensated by quickly delivered reflex activations (SLR) in the distal body segment. Transmitted via Ia afferent pathways, this has been associated with stiffening of the ankle joint complex leading to an improved recovery of posture in previous research (Granacher et al., 2006). To counteract the torque induced by posterior surface translation, an increase in SOL activation in SLR may have caused the decline in ankle joint velocity to regain stability of the body after unexpected surface translation (Hatzitaki et al., 2005; Granacher et al., 2006). At the same time, enhanced SOL activity without any changes in TA activity might be a result of a training-induced improved intermuscular coordination associated with a reduced antagonistic co-activation and a greater rate of force development (Behrens et al., 2015).

Concomitant to shank muscle activation, knee extensors (RF) were activated faster and more distinctly in early reflex phases (SLR and MLR) during marching in place perturbation. Augmented neuromuscular activation in the MLR and subsequent reflex responses (LLRs) are known to rely on the involvement of higher centers of the central nervous system such as the midbrain and brainstem (MLR) or even the motor cortex (LLRs) and thus, can generate specified reaction to the stimulus (Jacobs and Horak, 2007). Knee extensors are known to be predominantly activated in the eccentric phase of initial foot contact for impact absorption and/or energy storage in the elastic elements (Lacquaniti et al., 2012). Enhanced knee extensor activation, in terms of fall prevention, could lead to segmental stabilization (Horak and Nashner, 1986; Schillings et al., 2000; Gruber and Gollhofer, 2004), a consolidation of COM vertically above the support surface (Pfusterschmied et al., 2013), and might, therefore, finally reduce the risk of falling during slips and stumbles.

With an emphasis on the later reflex phases (120 ms after perturbation), next to muscle activation in SOL-RF, also TA and BF activities were raised for both groups. While early reflex responses are provided by Ia afferent pathways, supraspinal pathways are involved in later reflex responses (Taube et al., 2008). Thus, greatest muscle activation in later reflex phases might point toward a strategy induced by the motor cortex to counteract the perturbation stimulus by means of fall avoidance.

Phase- and segment-specific distinctions for neuromuscular control of postural responses after perturbation are manifested. Significant interaction effects and greater effect sizes for RBT versus CBT indicate intervention-dependent adaptations. Training-specific distinctions exist for RF activity, which are more pronounced during stance perturbation after RBT, a training that simulates fall characteristic situations associated with the following paragraph.

### Joint Kinematics and Multi-Segmental Strategy

The second aspect associated with fall avoidance deals with segmental joint kinematics. Despite the complex nature of falls, studies show that falls can be significantly reduced by reducing fall risk factors (Rubenstein, 2006). In the current investigation, enhanced muscle activation was accompanied by reduced angular velocity during stance (ankle joint) and marching in place perturbation (ankle and hip joint). Augmented ankle and hip joint velocities have been identified to be distinctive in fallers and vice versa, reduced hip and ankle joint velocities in non-fallers (Lee and Kerrigan, 1999; Lord et al., 2001). With reference the aforementioned articles, our results can be interpreted as follows: participants frequently exposed to the fall situation in training – as it occurs during RBT – may learn from the risk situation (Pai et al., 2010; Bhatt et al., 2011), adapting their motor behavior (Bhatt et al., 2011) by activating skeletal muscles appropriately to counteract postural deterioration and restrict joint movement and velocity (Cham and Redfern, 2002; Bhatt et al., 2006; Pai et al., 2010; Parijat and Lockhart, 2012; McCrum et al., 2014). Thereby, significantly elevated knee deflections – well pronounced after RBT and CBT – are in line with evidence during stance perturbation (Di Giulio et al., 2013) and slipping situations in locomotion (Cham and Redfern, 2001; Bhatt et al., 2006). Knee joint flexion led to a lowering of COM height and an immediate postural unloading of the perturbed foot (Sawer et al., 2017); both are associated with a rapid reacquisition of a stable COM during unpredictable slips known to be essential to secure postural control and to reduce fall risk (Schillings, 2005; Bhatt et al., 2006). Inter alia, a smaller knee deflection has been identified in patients with postural instabilities (Horak et al., 2005; Bakker et al., 2006) and is associated with enhanced joint rigidity (Chmielewski et al., 2005) and reduced range of motion (Hatzitaki et al., 2005).

These findings with greater effect sizes after RBT indicate that this training caused a pronounced shift in multi-segmental organization using a stiff ankle joint for immediate compensation, a deflected knee for the reacquisition of a stable COM, and reduced hip joint velocities to control COM and trunk movements to achieve fast balance recovery and safe body equilibrium.

### Distinction RBT and CBT and Functional Relevance

With neuromuscular control being the relevant aspect to determine the quality of postural response, adaptations of both interventions might be associated with a reduced fall incidence that could be relevant for fallers across the lifespan (Rubenstein, 2006; Bhatt and Pai, 2008; Granacher et al., 2010; Bieryla and Madigan, 2011). During stance perturbation, RBT as well as CBT enhanced RF activity and concomitantly reduced joint velocities in the proximal limb segment (knee and hip), but greater effects could be observed after RBT. This is in accordance with earlier investigations, emphasizing an intervention-specific adaptation due to specific training tasks (Freyler et al., 2016). The musculature of the proximal segments is well known to generate compensatory forces to restore equilibrium after slips and stumbles (Horak and Nashner, 1986; Hall and Jensen, 2002; Hatzitaki et al., 2005), and participants benefit from a two-segmental strategy to restore postural equilibrium and stabilize the trunk to prevent falling (Tang and Woollacott, 1998; Hall and Jensen, 2002; Schillings, 2005). During marching in place perturbation, early activation of distal *and* proximal activity is achieved after both training interventions, but effect sizes are even greater after CBT. Although this finding surprised us, comparable results for the shank musculature have been reported in more static paradigms in other studies (Gruber and Gollhofer, 2004; Taube et al., 2007; Freyler et al., 2016). Thereby, it can be supposed that CBT purposefully acts on the distal body segment (Freyler et al., 2016) making use of the knee joints that allows the stabilization of the COM within critical trajectories, and thus may reduce fall incidence (Pai et al., 2010; Bhatt et al., 2011).

### Limitations

For a conclusive statement, it is crucial to consider the limitations of the study. Even though the methodological approach in the current paper was carefully chosen based on previous evidence, three limiting aspects could not be ruled out.

First, fall avoidance has to be differentiated from fall incidence: The population investigated is known to have great skills regarding motor control. Thus, it is an ideal population to investigate fall avoidance during stumbling situations. However, actual fall incidences were not observed which is why a differentiation of kinematic and neuromuscular characteristics cannot be provided based on current results.

Second, simulation of stumbling executed in laboratory paradigms as close to everyday life situations as possible is always biased. For instance, we had to inform participants about possible stumbling situations due to ethical reasons. Thus, they knew that a fall risk situation could occur. However, participants were not aware about the mechanical attributes of the surface translation and the onset of the perturbation occurred randomly so that anticipatory muscle activity could still be excluded.

Third, we compared two – due to evidence – *effective* training regimens for postural control. Therefore, both trainings demonstrated improved neuromuscular and kinematic adaptations. The effectiveness of both trainings might be the reason for missing interaction effects in fastest reflex responses. Even though the comparison of RBT and CBT is restricted to the comparison of effect sizes, this could still point toward differential adaptations.

## Conclusion

Both training modalities improved reactive balance recovery after perturbations with adaptations being task-specific. Medium to large effect sizes were observed for neuromuscular and kinematic responses for RBT in response to stance perturbations, a task similar to the one which had been trained. This study provides basic evidence that neuromuscular control can be acquired rapidly by frequently reproducing the unexpected nature of real-life slipping situations within 4 weeks. It can be concluded that with repeated exposure to simulated slips, the central nervous system learns to choose a more effective muscle synergy and segmental organization to achieve fast balance recovery. Therefore, in dependence on the respective balance demands and independently of the stimulus itself, the participant can create a situation-specific postural stabilization strategy, and thus may have reduced the incidence of falling after RBT. While the current investigation is limited to neuromuscular adaptation in a healthy population, future studies across the lifespan might benefit from the current results by means of basic scientific evidence.

Conclusively, this study might set an essential cornerstone for further fall prevention investigations across the lifespan. Future investigations are needed which investigate, if especially high-risk fallers such as children and the elderly could benefit from RBT as a special form of CBT by counteracting age- or disease-associated degeneration of neuromuscular and kinematic strategies.

## Author Contributions

All authors made substantial contributions to the conception or design of the work, the acquisition, analysis, and interpretation of data for the work. Further they contributed drafting the work and revising it critically, they helped with the final approval of the version to be published and made the agreement to be accountable for all aspects of the work in ensuring that questions related to the accuracy or integrity of any part of the work are appropriately investigated and resolved.

## Conflict of Interest Statement

The authors declare that the research was conducted in the absence of any commercial or financial relationships that could be construed as a potential conflict of interest. The handling Editor declared a shared affliation, though no other collaboration, with one of the authors AK.

## References

[B1] Alexander B. H. Rivara F. P. Wolf M. E. (1992). The cost and frequency of hospitalization for fall-related injuries in older adults. *Am. J. Public Health* 82 1020–1023. 10.2105/AJPH.82.7.1020 1609903 PMC1694056

[B2] Arampatzis A. Karamanidis K. Mademli L. (2008). Deficits in the way to achieve balance related to mechanisms of dynamic stability control in the elderly. *J. Biomech.* 41 1754–1761. 10.1016/j.jbiomech.2008.02.022 18395209

[B3] Bakker M. Allum J. Visser J. Gruneberg C. Vandewarrenburg B. Kremer B. (2006). Postural responses to multidirectional stance perturbations in cerebellar ataxia. *Exp. Neurol.* 202 21–35. 10.1016/j.expneurol.2006.05.008 16808916

[B4] Behrens M. Mau-Moeller A. Wassermann F. Bader R. Bruhn S. (2015). Effect of balance training on neuromuscular function at rest and during isometric maximum voluntary contraction. *Eur. J. Appl. Physiol.* 115 1075–1085. 10.1007/s00421-014-308965-1 25557387

[B5] Bhatt T. Pai Y. C. (2008). Generalization of gait adaptation for fall prevention: from moveable platform to slippery floor. *J. Neurophysiol.* 101 948–957. 10.1152/jn.91004.2008 19073804 PMC2657073

[B6] Bhatt T. Wening J. D. Pai Y.-C. (2006). Adaptive control of gait stability in reducing slip-related backward loss of balance. *Exp. Brain Res.* 170 61–73. 10.1007/s00221-005-0189-5 16344930

[B7] Bhatt T. Yang F. Pai Y.-C. (2011). Learning from falling: retention of fall-resisting behavior derived from one episode of laboratory-induced slip training. *J. Am. Geriatr. Soc.* 59 2392–2393. 10.1111/j.1532-5415.2011.03708.x 22188094 PMC3390204

[B8] Bierbaum S. Peper A. Arampatzis A. (2013). Exercise of mechanisms of dynamic stability improves the stability state after an unexpected gait perturbation in elderly. *AGE* 35 1905–1915. 10.1007/s11357-012-9481-z 23054828 PMC3776125

[B9] Bieryla K. A. Madigan M. L. (2011). Proof of concept for perturbation-based balance training in older adults at a high risk for falls. *Arch. Phys. Med. Rehabil.* 92 841–843. 10.1016/j.apmr.2010.12.004 21530733

[B10] Bruhn S. Kullmann N. Gollhofer A. (2006). Combinatory effects of high-intensity-strength training and sensorimotor training on muscle strength. *Int. J. Sports Med.* 27 401–406. 10.1055/s-2005-865750 16729384

[B11] Cham R. Redfern M. S. (2001). Lower extremity corrective reactions to slip events. *J. Biomech.* 34 1439–1445. 10.1016/S0021-9290(01)00116-6 11672718

[B12] Cham R. Redfern M. S. (2002). Changes in gait when anticipating slippery floors. *Gait Posture* 15 159–171. 10.1016/S0966-6362(01)00150-3 11869910

[B13] Chmielewski T. L. Hurd W. J. Rudolph K. S. Axe M. J. Snyder-Mackler L. (2005). Perturbation training improves knee kinematics and reduces muscle co-contraction after complete unilateral anterior cruciate ligament rupture. *Phys. Ther.* 85 740–749; discussion 750–754. 16048422

[B14] Cohen J. (1988). *Statistical Power analysis for the Behavioral Sciences*, 2nd Edn Hillsdale, NJ: L. Erlbaum Associates.

[B15] Cohen J. (1992). A power primer. *Psychol. Bull.* 112 155–159. 10.1037/0033-2909.112.1.155 19565683

[B16] Di Giulio I. Baltzopoulos V. Maganaris C. N. Loram I. D. (2013). Human standing: does the control strategy preprogram a rigid knee? *J. Appl. Physiol.* 114 1717–1729. 10.1152/japplphysiol.01299.2012 23620493

[B17] Diener H. C. Horak F. B. Nashner L. M. (1988). Influence of stimulus parameters on human postural responses. *J. Neurophysiol.* 59 1888–1905. 10.1152/jn.1988.59.6.1888 3404210

[B18] Dietz V. Horstmann G. A. Berger W. (1989). Interlimb coordination of leg-muscle activation during perturbation of stance in humans. *J. Neurophysiol.* 62 680–693. 10.1152/jn.1989.62.3.680 2769353

[B19] Dobkin B. H. Dobkin B. H. (2003). *The Clinical Science of Neurologic Rehabilitation*, 2nd Edn New York, NY: Oxford University Press.

[B20] Faul F. Erdfelder E. Lang A.-G. Buchner A. (2007). G^∗^Power 3: a flexible statistical power analysis program for the social, behavioral, and biomedical sciences. *Behav. Res. Methods* 39 175–191. 10.3758/BF03193146 17695343

[B21] Fitzgerald G. K. Axe M. J. Snyder-Mackler L. (2000). The efficacy of perturbation training in nonoperative anterior cruciate ligament rehabilitation programs for physical active individuals. *Phys. Ther.* 80 128–140. 10654060

[B22] Fitzgerald G. K. Childs J. D. Ridge T. M. Irrgang J. J. (2002). Agility and perturbation training for a physically active individual with knee osteoarthritis. *Phys. Ther.* 82 372–382. 11922853

[B23] Freyler K. Gollhofer A. Colin R. Brüderlin U. Ritzmann R. (2015). Reactive balance control in response to perturbation in unilateral stance: interaction effects of direction, displacement and velocity on compensatory neuromuscular and kinematic responses. *PLoS One* 10:e0144529. 10.1371/journal.pone.0144529 26678061 PMC4683074

[B24] Freyler K. Krause A. Gollhofer A. Ritzmann R. (2016). Specific stimuli induce specific adaptations: sensorimotor training vs. reactive balance training. *PLoS One* 11:e0167557. 10.1371/journal.pone.0167557 27911944 PMC5135127

[B25] Freyler K. Weltin E. Gollhofer A. Ritzmann R. (2014). Improved postural control in response to a 4-week balance training with partially unloaded bodyweight. *Gait Posture* 40 291–296. 10.1016/j.gaitpost.2014.04.186 24836698

[B26] Gallagher S. S. Finison K. Guyer B. Goodenough S. (1984). The incidence of injuries among 87,000 Massachusetts children and adolescents: results of the 1980-81 statewide childhood injury prevention program surveillance system. *Am. J. Public Health* 74 1340–1347. 10.2105/AJPH.74.12.1340 6507685 PMC1652665

[B27] Ganley K. J. Powers C. M. (2005). Gait kinematics and kinetics of 7-year-old children: a comparison to adults using age-specific anthropometric data. *Gait Posture* 21 141–145. 10.1016/j.gaitpost.2004.01.007 15639392

[B28] Gehring D. Faschian K. Lauber B. Lohrer H. Nauck T. Gollhofer A. (2014). Mechanical instability destabilises the ankle joint directly in the ankle-sprain mechanism. *Br. J. Sports Med.* 48 377–382. 10.1136/bjsports-2013-092626 24124039

[B29] Gollhofer A. Rapp W. (1993). Recovery of stretch reflex responses following mechanical stimulation. *Eur. J. Appl. Physiol.* 66 415–420. 10.1007/BF00599614 8330609

[B30] Granacher U. Gollhofer A. (2005). Auswirkungen des Alterns auf die Schnellkraftfaehigkeit und das Reflexverhalten. / The impact of aging on explosive force production and on postural reflexes. *Dtsch. Z. Fuer Sportmed.* 56 68–73.

[B31] Granacher U. Gollhofer A. Kriemler S. (2010). Effects of balance training on postural sway, leg extensor strength, and jumping height in adolescents. *Res. Q. Exerc. Sport* 81 245–251. 10.1080/02701367.2010.10599672 20949844

[B32] Granacher U. Gollhofer A. Strass D. (2006). Training induced adaptations in characteristics of postural reflexes in elderly men. *Gait Posture* 24 459–466. 10.1016/j.gaitpost.2005.12.007 16472525

[B33] Granacher U. Gruber M. Gollhofer A. (2009). Auswirkungen von sensomotorischem training auf die posturale Kontrolle älterer Männer. *Dtsch. Z. Für Sportmed.* 60 387–393.

[B34] Granacher U. Muehlbauer T. Gollhofer A. Kressig R. W. Zahner L. (2011a). An intergenerational approach in the promotion of balance and strength for fall prevention - a mini-review. *Gerontology* 57 304–315. 10.1159/000320250 20720401

[B35] Granacher U. Muehlbauer T. Zahner L. Gollhofer A. Kressig R. W. (2011b). Comparison of traditional and recent approaches in the promotion of balance and strength in older adults. *Sports Med.* 41 377–400. 10.2165/11539920-000000000-00000 21510715

[B36] Granacher U. Muehlbauer T. Gruber M. (2012). A qualitative review of balance and strength performance in healthy older adults: impact for testing and training. *J. Aging Res.* 2012 1–16. 10.1155/2012/708905 22315687 PMC3270412

[B37] Grey M. J. Ladouceur M. Andersen J. B. Nielsen J. B. Sinkjaer T. (2001). Group II muscle afferents probably contribute to the medium latency soleus stretch reflex during walking in humans. *J. Physiol.* 534 925–933. 10.1111/j.1469-7793.2001.00925.x 11483721 PMC2278750

[B38] Gruber M. Gollhofer A. (2004). Impact of sensorimotor training on the rate of force development and neural activation. *Eur. J. Appl. Physiol.* 92 98–105. 10.1007/s00421-004-1080-y 15024669

[B39] Gruber M. Gruber S. B. H. Taube W. Schubert M. Beck S. C. Gollhofer A. (2007a). Differential effects of ballistic versus sensorimotor training on rate of force development and neural activation in humans. *J. Strength Cond. Res.* 21 274–282. 10.1519/R-20085.1 17313292

[B40] Gruber M. Taube W. Gollhofer A. Beck S. Amtage F. Schubert M. (2007b). Training-specific adaptations of H- and stretch reflexes in human soleus muscle. *J. Mot. Behav.* 39 68–78. 10.3200/JMBR.39.1.68-78 17251172

[B41] Halaki M. Gi K. (2012). “Normalization of EMG signals: to normalize or not to normalize and what to normalize to?,” in *Computational Intelligence in Electromyography Analysis - A Perspective on Current Applications and Future Challenges*, ed. Naik G. R. (Rijeka: InTech).

[B42] Hall C. D. Jensen J. L. (2002). Age-related differences in lower extremity power after support surface perturbations. *J. Am. Geriatr. Soc.* 50 1782–1788. 10.1046/j.1532-5415.2002.50505.x 12410895

[B43] Hatzitaki V. Amiridis I. G. Arabatzi F. (2005). Aging effects on postural responses to self-imposed balance perturbations. *Gait Posture* 22 250–257. 10.1016/j.gaitpost.2004.09.010 16214664

[B44] Heinrich S. Rapp K. Rissmann U. Becker C. König H.-H. (2010). Cost of falls in old age: a systematic review. *Osteoporos. Int.* 21 891–902. 10.1007/s00198-009-1100-1 19924496

[B45] Henry S. M. Fung J. Horak F. B. (1998). EMG responses to maintain stance during multidirectional surface translations. *J. Neurophysiol.* 80 1939–1950. 10.1152/jn.1998.80.4.1939 9772251

[B46] Hermens H. J. Freriks B. Disselhorst-Klug C. Rau G. (2000). Development of recommendations for SEMG sensors and sensor placement procedures. *J. Electromyogr. Kinesiol.* 10 361–374. 10.1016/S1050-6411(00)00027-4 11018445

[B47] Horak F. B. Dimitrova D. Nutt J. G. (2005). Direction-specific postural instability in subjects with Parkinson’s disease. *Exp. Neurol.* 193 504–521. 10.1016/j.expneurol.2004.12.008 15869953

[B48] Horak F. B. Nashner L. M. (1986). Central programming of postural movements: adaptation to altered support-surface configurations. *J. Neurophysiol.* 55 1369–1381. 10.1152/jn.1986.55.6.1369 3734861

[B49] Horstmann G. A. Dietz V. (1990). A basic posture control mechanism: the stabilization of the centre of gravity. *Electroencephalogr. Clin. Neurophysiol.* 76 165–176. 10.1016/0013-4694(90)90214-5 1697244

[B50] Hurd W. J. Chmielewski T. L. Snyder-Mackler L. (2006). Perturbation-enhanced neuromuscular training alters muscle activity in female athletes. *Knee Surg. Sports Traumatol. Arthrosc.* 14 60–69. 10.1007/s00167-005-0624-y 15937713

[B51] Jacobs J. V. Horak F. B. (2007). Cortical control of postural responses. *J. Neural. Transm.* 114 1339–1348. 10.1007/s00702-007-0657-0 17393068 PMC4382099

[B52] Karamanidis K. Arampatzis A. (2006). Age-related degeneration in leg-extensor muscle–tendon units decreases recovery performance after a forward fall: compensation with running experience. *Eur. J. Appl. Physiol.* 99 73–85. 10.1007/s00421-006-0318-2 17063362

[B53] Karlsson M. K. Vonschewelov T. Karlsson C. Coster M. Rosengen B. E. (2013). Prevention of falls in the elderly: a review. *Scand. J. Public Health* 41 442–454. 10.1177/1403494813483215 23554390

[B54] Lacquaniti F. Ivanenko Y. P. Zago M. (2012). Patterned control of human locomotion: control of human locomotion. *J. Physiol.* 590 2189–2199. 10.1113/jphysiol.2011.215137 22411012 PMC3424743

[B55] Lee L. W. Kerrigan D. C. (1999). Identification of kinetic differences between fallers and nonfallers in the elderly. *Am. J. Phys. Med. Rehabil.* 78 242–246. 10.1097/00002060-199905000-00011 10340422

[B56] Lesinski M. Hortobágyi T. Muehlbauer T. Gollhofer A. Granacher U. (2015). Dose-response relationships of balance training in healthy young adults: a systematic review and meta-analysis. *Sports Med.* 45 557–576. 10.1007/s40279-014-0284-5 25430598

[B57] Lord S. R. Sherrington C. Menz H. B. (2001). *Falls in Older People: Risk Factors and Strategies for Prevention*. Cambridge: Cambridge University Press.

[B58] Madureira M. M. Takayama L. Gallinaro A. L. Caparbo V. F. Costa R. A. Pereira R. M. R. (2007). Balance training program is highly effective in improving functional status and reducing the risk of falls in elderly women with osteoporosis: a randomized controlled trial. *Osteoporos. Int.* 18 419–425. 10.1007/s00198-006-0252-5 17089080 PMC1820755

[B59] Maki B. E. McIlroy M. E. (2006). Control of rapid limb movements for balance recovery: age-related changes and implications for fall prevention. *Age Ageing* 35 ii12–ii18. 10.1093/ageing/afl078 16926197

[B60] Mansfield A. Aqui A. Centen A. Danells C. J. DePaul V. G. Knorr S. (2015). Perturbation training to promote safe independent mobility post-stroke: study protocol for a randomized controlled trial. *BMC Neurol.* 15:87. 10.1186/s12883-015-0347-8 26048054 PMC4456796

[B61] Mansfield A. Peters A. L. Liu B. A. Maki B. E. (2010). Effect of a perturbation-based balance training program on compensatory stepping and grasping reactions in older adults: a randomized controlled trial. *Phys. Ther.* 90 476–491. 10.2522/ptj.20090070 20167644

[B62] McCrum C. Eysel-Gosepath K. Epro G. Meijer K. Savelberg H. H. Brüggemann G.-P. (2014). Deficient recovery response and adaptive feedback potential in dynamic gait stability in unilateral peripheral vestibular disorder patients. *Physiol. Rep.* 2:e12222. 10.14814/phy2.12222 25501424 PMC4332206

[B63] Miller T. R. Romano E. O. Spicer R. S. (2000). The cost of childhood unintentional injuries and the value of prevention. *Future Child.* 10 137–163. 10.2307/1602828 10911691

[B64] Mornieux G. Gehring D. Tokuno C. Gollhofer A. Taube W. (2014). Changes in leg kinematics in response to unpredictability in lateral jump execution. *Eur. J. Sport Sci.* 14 678–685. 10.1080/17461391.2014.894577 24621298

[B65] Nagai K. Yamada M. Tanaka B. Uemura K. Mori S. Aoyama T. (2012). Effects of balance training on muscle coactivation during postural control in older adults: a randomized controlled trial. *J. Gerontol. A Biol. Sci. Med. Sci.* 67 882–889. 10.1093/gerona/glr252 22389467

[B66] Obuchi S. Kojima M. Shiba Y. Shimada H. Suzuki T. (2004). A randomized controlled trial of a treadmill training with the perturbation to improve the balance performance in the community dwelling elderly subjects. *Nihon Ronen Igakkai Zasshi* 41 321–327. 10.3143/geriatrics.41.321 15237752

[B67] Oliveira A. S. C. Brito Silva P. Farina D. Kersting U. G. (2013). Unilateral balance training enhances neuromuscular reactions to perturbations in the trained and contralateral limb. *Gait Posture* 38 894–899. 10.1016/j.gaitpost.2013.04.015 23706505

[B68] Pai Y.-C. Bhatt T. Wang E. Espy D. Pavol M. J. (2010). Inoculation against falls: rapid adaptation by young and older adults to slips during daily activities. *Arch. Phys. Med. Rehabil.* 91 452–459. 10.1016/j.apmr.2009.10.032 20298839 PMC2842602

[B69] Parijat P. Lockhart T. E. (2012). Effects of moveable platform training in preventing slip-induced falls in older adults. *Ann. Biomed. Eng.* 40 1111–1121. 10.1007/s10439-011-0477-0 22134467 PMC3319506

[B70] Pfusterschmied J. Stöggl T. Buchecker M. Lindinger S. Wagner H. Müller E. (2013). Effects of 4-week slackline training on lower limb joint motion and muscle activation. *J. Sci. Med. Sport* 16 562–566. 10.1016/j.jsams.2012.12.006 23333134

[B71] Rubenstein L. Z. (2006). Falls in older people: epidemiology, risk factors and strategies for prevention. *Age Ageing* 35 ii37–ii41. 10.1093/ageing/afl084 16926202

[B72] Sawers A. Pai Y. C. Bhatt T. Ting L. H. (2017). Neuromuscular responses differ between slip-induced falls and recoveries in older adults. *J. Neurophysiol.* 117 509–522. 10.1152/jn.00699.2016 27832608 PMC5288485

[B73] Schillings A. M. (2005). Stumbling over obstacles in older adults compared to young adults. *J. Neurophysiol.* 94 1158–1168. 10.1152/jn.00396.2004 15615837

[B74] Schillings A. M. van Wezel B. M. Mulder T. Duysens J. (2000). Muscular responses and movement strategies during stumbling over obstacles. *J. Neurophysiol.* 83 2093–2102. 10.1152/jn.2000.83.4.2093 10758119

[B75] Schmitt D. (2003). Insights into the evolution of human bipedalism from experimental studies of humans and other primates. *J. Exp. Biol.* 206 1437–1448. 10.1242/jeb.00279 12654883

[B76] Shimada H. Obuchi S. Furuna T. Suzuki T. (2004). New intervention program for preventing falls among frail elderly people: the effects of perturbed walking exercise using a bilateral separated treadmill. *Am. J. Phys. Med. Rehabil.* 83 493–499. 10.1097/01.PHM.0000130025.54168.91 15213472

[B77] Shumway-Cook A. Hutchinson S. Kartin D. Price R. Woollacott M. (2003). Effect of balance training on recovery of stability in children with cerebral palsy. *Dev. Med. Child Neurol.* 45 591–602. 10.1111/j.1469-8749.2003.tb00963.x 12948326

[B78] Stevens J. A. Corso P. S. Finkelstein E. A. Miller T. R. (2006). The costs of fatal and non-fatal falls among older adults. *Inj. Prev* 12 290–295. 10.1136/ip.2005.011015 17018668 PMC2563445

[B79] Tang P. F. Woollacott M. H. (1998). Inefficient postural responses to unexpected slips during walking in older adults. *J. Gerontol. A Biol. Sci. Med. Sci.* 53 M471–M480. 10.1093/gerona/53A.6.M471 9823752

[B80] Taube W. (2006). Direct corticospinal pathways contribute to neuromuscular control of perturbed stance. *J. Appl. Physiol.* 101 420–429. 10.1152/japplphysiol.01447.2005 16601305

[B81] Taube W. Gruber M. Gollhofer A. (2008). Spinal and supraspinal adaptations associated with balance training and their functional relevance. *Acta Physiol.* 193 101–116. 10.1111/j.1748-1716.2008.01850.x 18346210

[B82] Taube W. Kullmann N. Leukel C. Kurz O. Amtage F. Gollhofer A. (2007). Differential reflex adaptations following sensorimotor and strength training in young elite athletes. *Int. J. Sports Med.* 28 999–1005. 10.1055/s-2007-964996 17497570

[B83] Thalheimer W. Cook S. (2002). *How to Calculate Effect Sizes from Published Research Articles: A Simplified Methodology*. Available at: http://www.bwgriffin.com/gsu/courses/edur9131/content/Effect_Sizes_pdf5.pdf [accessed November 30, 2016].

[B84] Timsina L. R. Willetts J. L. Brennan M. J. Marucci-Wellman H. Lombardi D. A. Courtney T. K. (2017). Circumstances of fall-related injuries by age and gender among community-dwelling adults in the United States. *PLoS One* 12:e0176561. 10.1371/journal.pone.0176561 28472065 PMC5417511

[B85] Tinetti M. E. (1994). Prevention of falls and fall injuries in elderly persons: a research agenda. *Prev. Med.* 23 756–762. 10.1006/pmed.1994.1130 7845954

[B86] Ungar A. Rafanelli M. Iacomelli I. Brunetti M. A. Ceccofiglio A. Tesi F. (2013). Fall prevention in the elderly. *Clin. Cases Miner. Bone Metab.* 10 91–95. 24133524 PMC3797008

[B87] van Dieën J. H. Pijnappels M. Bobbert M. F. (2005). Age-related intrinsic limitations in preventing a trip and regaining balance after a trip. *Saf. Sci.* 43 437–453. 10.1016/j.ssci.2005.08.008

[B88] Winter D. (1995). Human balance and posture control during standing and walking. *Gait Posture* 3 193–214. 10.1016/0966-6362(96)82849-9

[B89] Yim-Chiplis P. K. Talbot L. A. (2000). Defining and measuring balance in adults. *Biol. Res. Nurs.* 1 321–331. 10.1177/109980040000100408 11232210

