# Supplementary material for: Neuromuscular and Kinematic Adaptation in Response to Reactive Balance Training – a Randomized Controlled Study Regarding Fall Prevention
Source: Front Physiol. 2018 Aug 7;9:1075. doi: 10.3389/fphys.2018.01075 (PMC6090079; doi:10.3389/fphys.2018.01075)
Supplement: Supplementary file 1 [file Table_1.docx]

**Supplementary Table 1: Coefficient of variation for all muscles.**

Described are the values of coefficients of variance for the reflex phases short-latency (SLR), medium-latency (MLR) and long-latency response (LLR, LLR_120-150_, LLR_150-210_) for lower limb muscles rectus femoris (RF), biceps femoris (BF), tibialis anterior (TA) and soleus (SOL) after reactive balance training (RBT) and conventional balance training (CBT).

**Protocol 1: Stance perturbation**

| **SLR** | | **pre** | **post** | **difference** |
| --- | --- | --- | --- | --- |
| **RF** | **RBT** | .55 | .67 | + .12 |
|  | **CBT** | 1.44 | .63 | - .81 |
| **BF** | **RBT** | .48 | 1.34 | + .87 |
|  | **CBT** | .41 | 1.48 | +1.07 |
| **TA** | **RBT** | 1.21 | 1.11 | - .10 |
|  | **CBT** | .66 | .42 | - .23 |
| **SOL** | **RBT** | .69 | .58 | - .11 |
|  | **CBT** | .71 | .53 | - .18 |
| **MLR** | | **pre** | **post** | **difference** |
| **RF** | **RBT** | .58 | .61 | + .03 |
|  | **CBT** | 1.35 | .97 | - .38 |
| **BF** | **RBT** | .44 | 1.47 | +1.03 |
|  | **CBT** | .45 | 1.52 | +1.07 |
| **TA** | **RBT** | .90 | 1.41 | + .51 |
|  | **CBT** | .54 | .46 | - .07 |
| **SOL** | **RBT** | .56 | .54 | - .02 |
|  | **CBT** | .92 | .61 | - .31 |
| **LLR** | | **pre** | **post** | **difference** |
| **RF** | **RBT** | .74 | .70 | - .05 |
|  | **CBT** | .86 | 1.26 | + .40 |
| **BF** | **RBT** | .91 | 1.69 | + .79 |
|  | **CBT** | .48 | 1.93 | +1.45 |
| **TA** | **RBT** | 1.00 | 1.28 | + .28 |
|  | **CBT** | .67 | .41 | - .26 |
| **SOL** | **RBT** | .60 | .53 | - .08 |
|  | **CBT** | .65 | .63 | - .02 |
| **LLR_120-150_** | | **pre** | **post** | **difference** |
| **RF** | **RBT** | .81 | .85 | + .05 |
|  | **CBT** | 1.12 | .52 | - .53 |
| **BF** | **RBT** | .48 | .84 | + .76 |
|  | **CBT** | .77 | .75 | - .03 |
| **TA** | **RBT** | 1.38 | 1.02 | - .26 |
|  | **CBT** | .87 | 1.04 | + .19 |
| **SOL** | **RBT** | .68 | .52 | - .23 |
|  | **CBT** | .35 | .69 | + .95 |
| **LLR_150-210_** | | **pre** | **post** | **difference** |
| **RF** | **RBT** | .73 | 1.12 | + .54 |
|  | **CBT** | .99 | .66 | - .34 |
| **BF** | **RBT** | .50 | 1.21 | +1.41 |
|  | **CBT** | .85 | .89 | + .06 |
| **TA** | **RBT** | 1.26 | 1.22 | - .03 |
|  | **CBT** | .77 | .57 | - .27 |
| **SOL** | **RBT** | .74 | .92 | + .24 |
|  | **CBT** | .64 | .64 | - .01 |

**Protocol 2: Marching in place perturbation**

| **SLR** | | **pre** | **post** | **difference** |
| --- | --- | --- | --- | --- |
| **RF** | **RBT** | .53 | .54 | + .02 |
|  | **CBT** | 1.02 | .48 | - .54 |
| **BF** | **RBT** | .52 | 1.73 | +1.21 |
|  | **CBT** | .35 | 1.50 | +1.15 |
| **TA** | **RBT** | .93 | 1.05 | + .12 |
|  | **CBT** | .97 | .57 | - .40 |
| **SOL** | **RBT** | .72 | .92 | + .19 |
|  | **CBT** | .88 | .56 | - .32 |
| **MLR** | | **pre** | **post** | **difference** |
| **RF** | **RBT** | .67 | .62 | - .06 |
|  | **CBT** | .82 | .35 | - .48 |
| **BF** | **RBT** | .36 | 1.22 | + .86 |
|  | **CBT** | .40 | .24 | - .16 |
| **TA** | **RBT** | .88 | 1.55 | + .68 |
|  | **CBT** | .94 | .47 | - .48 |
| **SOL** | **RBT** | .63 | .86 | + .23 |
|  | **CBT** | .60 | .60 | + .00 |
| **LLR** | | **pre** | **post** | **difference** |
| **RF** | **RBT** | .66 | .76 | + .09 |
|  | **CBT** | .68 | .42 | - .26 |
| **BF** | **RBT** | .44 | 1.61 | +1.17 |
|  | **CBT** | .50 | 1.44 | + .95 |
| **TA** | **RBT** | 1.52 | 1.88 | + .36 |
|  | **CBT** | .72 | .50 | - .22 |
| **SOL** | **RBT** | .55 | .43 | - .13 |
|  | **CBT** | .82 | .62 | - .20 |
| **LLR_120-150_** | | **pre** | **post** | **difference** |
| **RF** | **RBT** | .92 | .79 | - .14 |
|  | **CBT** | .86 | .77 | - .11 |
| **BF** | **RBT** | .51 | 1.22 | +1.38 |
|  | **CBT** | .54 | 1.30 | +1.40 |
| **TA** | **RBT** | .89 | 1.36 | + .53 |
|  | **CBT** | .82 | .52 | - .37 |
| **SOL** | **RBT** | .67 | .48 | - .29 |
|  | **CBT** | .57 | .50 | - .13 |
| **LLR_150-210_** | | **pre** | **post** | **difference** |
| **RF** | **RBT** | 1.64 | .69 | - .58 |
|  | **CBT** | .90 | 1.38 | + .54 |
| **BF** | **RBT** | .63 | 1.53 | +1.42 |
|  | **CBT** | .68 | 1.28 | + .89 |
| **TA** | **RBT** | 1.13 | 1.00 | - .12 |
|  | **CBT** | 1.03 | .50 | - .52 |
| **SOL** | **RBT** | .71 | .83 | + .17 |
|  | **CBT** | .80 | 1.00 | + .26 |
