# Supplementary material for: Neuromuscular and Kinematic Adaptation in Response to Reactive Balance Training – a Randomized Controlled Study Regarding Fall Prevention
Source: Front Physiol. 2018 Aug 7;9:1075. doi: 10.3389/fphys.2018.01075 (PMC6090079; doi:10.3389/fphys.2018.01075)
Supplement: Supplementary file 2 [file Table_2.docx]

**Supplementary Table 2: Onset latency (absolute data) of the electromyograms during stance and marching in place perturbation.**

Depicted are absolute values for muscle activity onset (*±* standard deviations) after perturbation stimulus prior (pre) and after reactive balance training (RBT) or conventional balance training (CBT). ANOVAs describe statistical analysis of latency differences between muscles.

***Protocol 1: Stance perturbation - latencies [ms]***

|  | ***EMG*** | ***pre*** | ***post*** | ***changes*** *[%]* |
| --- | --- | --- | --- | --- |
| ***RBT*** | ***RF*** | *54 ± 15* | *46 ± 16* | *-15* |
|  | ***BF*** | *55 ± 19* | *49 ± 17* | *-11* |
|  | ***SOL*** | *50 ± 9* | *47 ± 8* | *-5* |
|  | ***ANOVA*** | ***F(4,90)=1.22, P=.31*** | ***F(4,90)= .35, P= .85*** |  |
|  | ***EMG*** | ***pre*** | ***post*** | ***changes*** *[%]* |
| ***CBT*** | ***RF*** | *48 ± 14* | *47 ± 14* | *-2* |
|  | ***BF*** | *51 ± 14* | *53 ± 15* | *+4* |
|  | ***SOL*** | *52 ± 12* | *46 ± 12* | *-12* |
|  | ***ANOVA*** | ***F(4,85)= .22, P=.93*** | ***F(4,85)= .89, P= .48*** |  |

***Protocol 2: Marching in place perturbation - Latencies [ms]***

|  | ***EMG*** | ***pre*** | ***post*** | ***changes*** *[%]* |
| --- | --- | --- | --- | --- |
| ***RBT*** | ***RF*** | *51 ± 13* | *44 ± 12* | *-14* |
|  | ***BF*** | *54 ± 20* | *46 ± 15* | *-14* |
|  | ***SOL*** | *52 ± 11* | *51 ± 9* | *-2* |
|  | ***ANOVA*** | ***F(4,90)= .35, P= .84*** | ***F(4,90)= 1.09, P= .37*** |  |
|  | ***EMG*** | ***pre*** | ***post*** | ***changes*** *[%]* |
| ***CBT*** | ***RF*** | *49 ± 14* | *48 ± 12* | *-3* |
|  | ***BF*** | *46 ± 10* | *49 ± 13* | *+8* |
|  | ***SOL*** | *48 ± 10* | *49 ± 11* | *+1* |
|  | ***ANOVA*** | ***F(4,85)= .63, P= .64*** | ***F(4,85)= .22, P = .93*** |  |
